# Knock-Down the Expression of Brassinosteroid Receptor *TaBRI1* Reduces Photosynthesis, Tolerance to High Light and High Temperature Stresses and Grain Yield in Wheat

**DOI:** 10.3390/plants9070840

**Published:** 2020-07-03

**Authors:** Jingjing Fang, Weiqi Zhu, Yiping Tong

**Affiliations:** 1 Institute of Genetics and Developmental Biology, Innovation Academy for Seed Design, Chinese Academy of Sciences, Beijing 100101, China; fangjingjing@caas.cn; 2 National Key Facility for Crop Gene Resources and Genetic Improvement, Institute of Crop Science, Chinese Academy of Agricultural Sciences, Beijing 100081, China; 3 The Zhao Xian Experimental Station of Hebei Academy of Agriculture and Forestry Sciences, Shijiazhuang 050035, China; zwqkyj@163.com

**Keywords:** *TaBRI1*, photosynthesis, high light and high temperature stresses, grain yield, wheat

## Abstract

Brassinosteroid (BR)-deficient or -insensitive mutants exhibited altered plant architecture with the potential to impact yield, the underlying physiological and molecular mechanisms are still to be explored. In this study, we cloned three BR receptor homologous genes *TaBRI1-A1*, *-B1* and *-D1* from hexaploid wheat (*Triticum estivum* L.) and further isolated the *TaBRI1-A1*, *TaBRI1-D1* deletion mutants from the ion beam-induced mutants of variety Xiaoyan81, *TaBRI1-A1* and *TaBRI1-D1* in which the expression of total receptor *TaBRI1* was significantly decreased. The *TaBRI1* knock-down mutants exhibited relatively erect leaves and a significant decrease in the 1000-grain weight. Further studies showed that *TaBRI1* knock-down mutants showed a significant reduction in photosynthetic rate during the whole grain-filling stage. *TaBRI1* knock-down plants generated by *TaBRI1-A1*, *TaBRI1-D1* deletion or using virus-induced gene silencing exhibited the reduction in the efficiency of photosystem II (PSII) (*Fv*/*Fm*, Φ_PSII_ and electron transport rate, ETR) especially under high light and high temperature stresses. The 24-epibrassinolide (EBR) treatment increased CO_2_ assimilation rate in the wild type under both normal and high light and high temperature stresses conditions, but this increasing effect was not observed in the *TaBRI1* knock-down mutants. Meanwhile, the expression levels of BR biosynthetic genes including *TaDWARF4*, *TaCPD1* and *TaCPD90C1* is not decreased or decreased to a lesser extent in the *TaBRI1* knock-down mutants after EBR treatment. These results suggested that *TaBRI1* is required for maintaining photosynthesis and tolerance to high light and high temperature stresses both of which are important for grain yield and will be a possible engineered target to control plant photosynthesis and yields in wheat.

## 1. Introduction

Wheat is one of the most important crops in the world. Great efforts have been made to enhance its productivity and quality to meet the 1.6% increase of demand for wheat per year [1]. Plant hormone, brassinosteroids (BRs), play crucial roles in modulating plant architecture and seed yield. Treatment of rice plantlets with 24-epibrassinolide (EBR) leads to a 22% increase in seed fresh weight per plant [2]. In addition, brassinolide (BR) treatment promotes plant growth rate, root size and dry weight of root and stem [3]. BRs regulate plant development and gene expression through receptor kinase-mediated signal transduction pathway [4].

BR signaling pathway is well established in both *Arabidopsis* and rice in recent years and it was suggested that rice has a conserved BR primary signaling pathway with that of *Arabidopsis* [5]. In brief, BRs are perceived at the plasma membrane by the leucine-rich repeat (LRR) receptor kinase BRI1 (brassinosteroid insensitive 1) [6]. Binding of BRs induces BRI1 dissociation of the inhibitory protein BKI1 (BR kinase inhibitor 1), BRI1 autophosphorylation and association/transphosphorylation with co-receptor BAK1 which lead to the accumulation of non-phosphorylated BZR1 and BZR2/BES1 in the nucleus through a series of phosphorylation and dephosphorylation processes [7,8,9,10]. The non-phosphorylated BZR1 and BZR2/BES1 further directly or indirectly activates the expression of BR-responsive genes, thus regulating plant growth and development [11].

In addition to the functions in plant growth and development, BRs also play prominent roles in plant stress responses to various abiotic stresses such as low and high temperature, drought, salinity, organic pollutant and heavy metal toxicity [12,13,14,15,16]. The onset of abiotic stresses severely affects photosynthesis as the stresses lead to the over-reduction of the electron transport chain (ETC) which, in turn, results in photo-oxidation [17,18]. BRs have been shown to increase photosynthetic capacity by activating Rubisco activase. BR treatment activates antioxidant machineries in plants which scavenge the toxic reactive oxygen species (ROS) to protect plants from ROS damage [19,20,21].

Manipulation of BR signaling further revealed the potential role of BRs in plant responses to abiotic stresses. The negative regulator BIN2 and key transcription factors BZR2/BES1 are operated to regulate different stress responsive transcription factors which activate stress adaptive signaling pathways [22]. BR signaling is also implicated in salt and cold stress responses via BRI1 [23,24]. Previous studies showed the salt hypersensitivity of *bri1-9* mutants could be partially rescued by inhibiting the endoplasmic reticulum-associated protein degradation system, suggesting that a membrane-bound BRI1 signaling complex is involved in the salinity response [23]. In addition, The *bri1-9* mutant generates an increased tolerance to cold stress due to it could always be alert to stresses by constitutive activation of subsets of defense [24]. Taken together, these findings provide evidence that the abiotic stresses were regulated by BR receptors and downstream signaling components.

Accordingly, plant growth would be modified by manipulation of endogenous BR content or BR signaling. In most cases, BR-deficient or -insensitive mutants displayed dwarfism, erect leaf, delayed flowering, reduced fertility and grain length [6,25,26,27,28]. However, suppression of BR related genes involved in the biosynthesis and signaling pathways with a slight level could significantly enhance the grain yield in plant [29,30]. For example, CYP90B1/DWARF4 catalyzed the rate-limiting step of brassinosteroid biosynthesis, C-22 hydroxylation. The biomass and grain yield in *osdwarf4-1* mutants are increased by nearly 40% and 26% compared with those in WT under high-density planting conditions, as the mutants show erect leaves and normal reproductive development [31]. The biomass of *d61-7*, a weak mutant of *OsBRI1*, increase by 35% in biomass than wild type at a high planting density [32]. Moreover, partially suppression of endogenous *OsBRI1* causes a 30% increase in grain yield, compared with wild type at high density [32]. Plant height is another important agronomic trait that directly affects crop architecture and grain yields. Barley mutant of *HvBRI1*, *uzu*, exhibited a semi-dwarf, upright plant architecture [33]. The *uzu* gene is currently being introduced into all of the hull-less cultivars in Japan which is very suitable for high yields as its semi-dwarf phenotype is in favor of lodging resistance. Additionally, numerous *BRI1* loss-of-function mutants have been identified in several species as well as *Arabidopsis*, including tomato (*Lycopersicon esculentum*), pea (*Pisum sativum*), rice (*Oryza sativa*) and *Brachypodium distachyon* [6,34,35,36]. These mutants exhibited similar pleiotropic phenotypes such as dwarfism, dark green leaves and photomorphogenesis in the dark which suggest that BRI1-mediated BR pathway are conserved in the plant development.

However, it has been reported that BR-deficient or -insensitive mutants exhibited altered plant architecture with the potential to impact yield, the underlying physiological mechanisms were remained unclear. Therefore, the main objectives of this study were (i) to examine the effects of deletion in *BRI1* homologous genes on plant architecture and grain yield in wheat and (ii) to detect the putative biologic functions of *TaBRI1* in regulating plant architecture and grain yield by measuring photosynthetic rate and assessing the tolerance ability to high light and high temperature stresses in *TaBRI1* deletion mutants. This study provides substantial information for understanding the function of *TaBRI1* in wheat.

## 2. Materials and Methods

### 2.1. Plant Materials and Growth Conditions

Common wheat (*Triticum estivum* L.) cv. Xiaoyan81 and *TaBRI1* mutants were used in this study. The mutants were obtained from Xiaoyan81 deletion mutant library constructed by nitrogen ion irradiation [37]. Xiaoyan81 is a winter wheat (*Triticum estivum*) variety which was released commercially in 2006.

Two connective field trials were conducted in 2013–2014 and 2014–2015 growing seasons at the Experimental Station of Institute of Genetics and Developmental Biology, Chinese Academy of Sciences, Beijing. The seeds of each material were grown in randomized blocks with three replications. The additives 18.0 g m^−2^ nitrogen (N) in the form of urea and 9.0 g m^−2^ phosphorus (P) as calcium superphosphate was supplied to the soil before sowing, and 5.2 g m^−2^-N was top-dressed at stem elongation stage. For each material in each replicate, 1000 seeds were sown in the 1.1 × 5 m plot with six rows and the rows were spaced 15 cm apart. The plants were supplied with sufficient water to avoid potential drought stress throughout the growing season and sprayed with fungicide to prevent foliar diseases.

Hydroponic culture was used to evaluate the seedling phenotypes of *TaBRI1* mutants. Hydroponic plants are also used for EBR treatment, analyzing the expression levels of *TaBRI1* and BR biosynthetic genes, and evaluating the tolerance of wheat seedlings to high light and high temperature (HLHT) stresses. The seeds were surface sterilized and germinated on a sterile filter paper soaked with distilled water for 7 days at 20 °C. Subsequently, 24 seedlings were transferred to 25 × 55 cm plastic boxes containing 14 L nutrient solution. The nutrient solution was composed as described by Ren et al. (2012) [38]. This solution was renewed every 3 days. The plants were grown in a controlled-environment growth chamber (HP-1000GS, Wuhan Ruihua Instrument & Equipment Co., Ltd., Wuhan, China) with the following conditions: 14-h-light/10-h-dark photoperiod at 350-μmol m^−2^ s^−1^ photosynthetic photon flux density (PPFD) under 20 °C with relative humidity of 60–70%.

### 2.2. Gene Cloning and Phylogenetic Analysis

Using the protein sequence of rice BRI1 published in GenBank (AP014957.1) to blast wheat EST library, the obtained 50 ESTs were used to assembly the complete *TaBRI1* sequence. Primers *TaBRI1(F)* and *TaBRI1(R3)* were designed at 5′and 3′ ends, respectively to amplify the full length of *TaBRI1* using Xiaoyan81 genomic DNA as template. The 3′-RACE PCR primer *TaBRI1(RACE)F1* were designed according to the identified ESTs. According to the 3′-RACE results, the specific primers *TaBRI1*.*1specific(R)*, *TaBRI1*.*2specific(R)* and *TaBRI1.3specific(R)* were designed on the 3′ UTR which were used to confirm the chromosome location of the genes and amplify the full length of *TaBRI1-A1*, *-D1* and *-B1*, respectively.

A phylogenetic tree of *TaBRI1* was generated (using the same software) by the neighbor-joining (NJ) method with Poisson correction [39]. All positions which contain alignment gaps were removed by the NJ analyses with pairwise sequence comparisons. Bootstrap replication (1000 replications) was used to quantify statistical support for the nodes in the phylogenetic tree.

### 2.3. Screening for TaBRI1-A1, TaBRI1-D1 Deletion Mutants

Genomic DNA samples were prepared from 5600 seedlings grown from randomly selected M_2_ seeds for screening the deletion mutants of *TaBRI1*. *TaBRI1*.*1(specific)R* (A chromosome specific primers) and *TaBRI1*.*2(specific)R* (D chromosome specific primers) obtained from 3′-RACE were mixed together with *TaBRI1(RACE)F1* to amplify using DNA of Xiaoyan81 ion beam mutagenesis population as template. The above primer sets amplified two fragments with different sizes, which were specific for the *TaBRI1-A1* (519 bp) and *TaBRI1-D1* (555 bp) genes of Xiaoyan81, respectively. According to the sizes of amplified fragments, deletion mutants of *TaBRI1* on chromosome *A* and chromosome *D* were obtained, respectively.

### 2.4. Vector Construction and Transformation

The coding fragments of *TaBRI1-A1*, *-B1* and *-D1* were amplified with primers *TaBRI1(pri)F* and *TaBRI1(pri)R* using the obtained *TaBRI1-A1*, *-B1* and *-D1* complete genomic sequence as templates, due to there are no introns for *TaBRI1* genomic sequence. The fragments were inserted into pEASY-blunt vector and digested with NdeI and BamHI. The fragment was then inserted into the pRI-101 vector by digesting with NdeI and BamHI. *bri1-5* mutants were used as receptor plants for transformation using the floral dip method. Seeds of wild-type (WT) Col-0, *bri1-5* and overexpression lines in *bri1-5* mutant background were germinated on half-strength Murashige and Skoog medium and transferred into soil for continuing growth at 22 °C under a 16-h-light/8-h-dark photoperiod.

### 2.5. Leaf Angle Measurements

The leaf angle during the grain-filling stage was measured with a protractor. The measurements were made in one plant at several different stages after an-thesis. Data were collected from fifteen flag leaves that were fully expanded in the center of each plot at every stage. The second-leaf angle at the seedling stage was measured with the method using three-week old seedlings.

### 2.6. Gas Exchange Measurements

CO_2_ gas exchange of leaves was measured with a portable LI-6400 photosynthesis system (LiCor-6400; LiCor, Inc., Lincoln, NE, USA) during post-anthesis period. The LI-6400 was operated as an open system. The measurement conditions were 450-µmol s^−1^ CO_2_ in the leaf chamber, 1200 µmol.m^−2^ s^−1^ PPFD and 25 °C of the temperature. Eight fully expanded flag leaves in the center of each plot were measured from 9:00 to 11:00 in the morning on a sunny day.

To evaluate the tolerance of wheat seedlings to HLHT stresses, WT and *TaBRI1* mutants were hydroponic cultured for 12 days according to the above method described in Section 2.1. The uniform plants were selected and then were exposed to white light provided by a halogen lamp (Flecta, Frankfurt, Germany). The leaves were exposed constantly to high light (1500-µmol m^−2^ s^−1^ PPFD on the leaf surface) under high temperature (35 ± 1 °C) with a relative humidity 60% for 3 h. Leaves were equilibrated for at least 3 min and CO_2_ assimilation rate (A), stomatal conductance (Gs), transpiration rate (E) and intercellular CO_2_ concentration (Ci) were recorded at a CO_2_ concentration of 380-µmol m^−2^ s^−1^ and at a temperature 22 °C with a relative humidity 60% and a light intensity of 500 µmol m^−2^ s^−1^ before and after the treatment.

### 2.7. Virus-Induced Gene Silencing (VIGS) Assay

To construct recombinant *BSMV:TaBRI1* vector, the 258 bp cDNA fragments of *TaBRI1* were amplified using primers 5′-AGCTAGCCAGAGCTTGGTTCTC-3′ and 5′- TGCTAGCGGTACTTGCCTCATC-3′ and subcloned in an antisense orientation into the Nhe I restriction site of the RNAγ of BSMV. For in vitro transcription, the *BSMVɑ*, *BSMV:GFP* and *BSMV:TaBRI1* vectors were linearized with Mlu I, and *BSMVβ* with SpeI which were used as templates to synthesize RNAs with the RiboMAXTM large scale RNA production system (Promega, Madison, WI, USA). The α, β, γ RNAs were mixed in equal amounts and then diluted with GKP buffer (0.05-mol/L glycine, 0.03-mol/L K_2_HPO4, 1% *w*/*v* bentonite and 1% *w*/*v* Celite at pH 9.2). The inoculation of each viral construct was performed according to previously described [40]. Briefly, the virus *BSMV:TaBRI1* and control virus *BSMV:GFP* were used to inoculate with the second fully expanded leaves from the bottom at 2-leaf stage. The incubated plants were grown at 90% relative humidity with darkness for 24 h at 23 ± 1 °C then grown under a 16-h -light/8-h-dark photoperiod.

### 2.8. Imaging Analysis of Chlorophyll Fluorescence

Flag leaves from WT and *TaBRI1* knock-down mutants were sampled at 21 days post-anthesis (DPA) and 28 DPA in the field. The flag leaves were detached, placed on moist gauze and then exposed to HLHT conditions supplied with high-light growth chamber (E36HO, Percival Scientific, Perry, IA, USA). The light reaching the leaf surface was 1500-µmol m^−2^ s^−1^ PPFD under 35 °C for 3 h with a relative humidity ca. 60%. The leaves were taken care to keep moist throughout the treatment. Chlorophyll fluorescence imaging of the flag leaves was measured using an imaging-PAM fluorometer (Walz, Effeltrich, Germany). Leaves were placed in darkness for 30 min prior to measurement. Maximum quantum yield of PSII (*Fv*/*Fm*), actual photochemical efficiency of PSII (Φ_PSII_) and electron transport rates (ETRs) were measured and calculated as described previously [41]. False-color images representing *Fv*/*Fm* and Φ_PSII_ levels in WT and mutant leaves were produced using the PAM software equipped with the imaging-PAM fluorometer.

### 2.9. RNA Extraction and Quantitative RT-PCR

Total RNA was extracted from wheat leaf samples using Trizol (Invitrogen, Waltham, MA, USA) and pretreated with DNase I. Two micrograms total RNA was used to synthesize the first-strand cDNA using Superscript III reverse transcriptase (Promega) following the manufacturer’s instructions. Using the protein sequence of rice *DWARF4*, *CPD1* and *CPD90D1* published in GenBank to blast wheat EST library, the obtained ESTs were used to assembly and primers were designed on the coding sequence (CDS). The SYBR Premix Ex Taq (TaKaRa; RR041A) was used to conduct the real-time PCR analysis on an ABI7900 HT fast real-time PCR system. cDNA product was used as template for 40 cycles of 95 °C for 30 s, 95 °C for 10 s and 60 °C for 30 s. The expression levels of *TaBRI1* and BR biosynthetic genes were normalized to *ACTIN1* gene and calculated using the 2^−∆∆CT^ method. RT-PCR was repeated at least 3 times for each sample and the used primers were listed in Table S1.

### 2.10. EBR Treatment

After hydroponic culturing for 12 days according to the above method Section 2.1, the uniform plants were selected and treated with 24-epibrassinolide (EBR). The seedling leaves were sprayed with 0.1-mg/L EBR until they were completely wet. The control leaves were sprayed with the same concentration of ethanol (1/1000, *v*/*v*) as the EBR was dissolved in ethanol when preparing the stock solution. After 48 h of EBR treatment, the seedlings were exposed to HLHT stresses for 3 h. The fully expanded second leaves were used for gas exchange measurements and gene expression analysis.

### 2.11. Statistical Analysis

All data in this study were presented as mean ± standard error (SE) or deviation (SD). Statistically significant differences was tested with SPSS software at *p* = 0.05 using ANOVA, post hoc LSD.

## 3. Results

### 3.1. Cloning of Three TaBRI1 Homologous Genes and the Expression Pattern of TaBRI1

**Figure 1:**
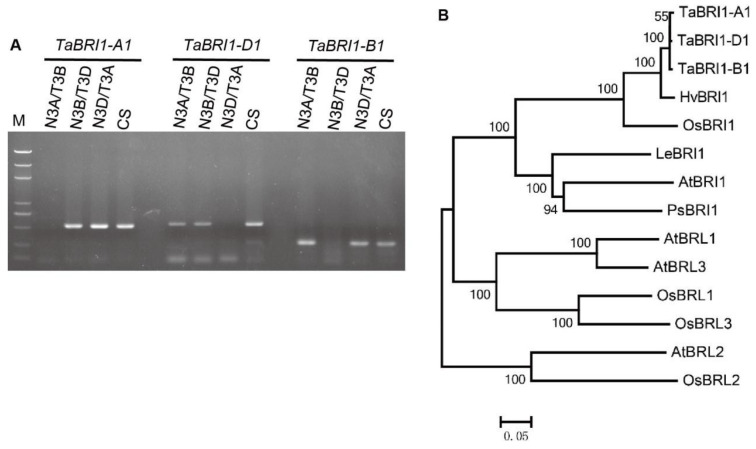
Chromosome location of *TaBRI1* and phylogenetic analysis. (**A**) Chromosome location of *TaBRI1* using nulli-tetrasomic and double ditelosomic lines derived from wheat cultivar Chinese Spring (CS). M, DL2000 DNA marker; N3A/T3B, N3B/T3D, N3D/T3A, three nulli-tetrasomic lines; (**B**) phylogenetic analysis of protein sequences of *TaBRI1* and other BRI1 from barley, rice, tomato, pea and *Arabidopsis*. The numbers on the branches presented refer to the degree of shared sequence similarity. The comparison included the following BRI1 family members: for barley, HvBRI1 (BAD06331); for rice, OsBRI1 (BAB68053), OsBRL1 (BAD34326), OsBRL2 (AAK52544) and OsBRL3 (BAD01717); for tomato, LeBRI1 (AAN85409); for pea, PsBRI1 (BAC99050); and for *Arabidopsis*, AtBRI1 (AAC49810), AtBRL1 (Q9ZWC8), AtBRL2 (Q9ZPS9) and AtBRL3 (Q9LJF3).

The full-length cDNA and genomic sequences of *TaBRI1* were obtained through 3′-RACE (rapid amplification of cDNA end) PCRs and genomic amplified, respectively, from the winter wheat variety Xiaoyan81. Comparison of the genomic DNA and cDNA sequences revealed that the *TaBRI1* gene structure contains no introns and had large variations in the 3′-UTRs. Use the gene specific primers located at the 3′-UTRs and the Chinese Spring deletion lines to identify the chromosome location of the genes, the results showed that these three *TaBRI1* genes were mapped on chromosomes 3A, 3B and 3D (Figure 1A). Phylogenetic analysis indicated that the three *TaBRI1 s* fall into the same clade with OsBRI1 of rice and HvBRI1 of barley and were closely related to HvBRI1 (Figure 1B).

**Figure 2:**
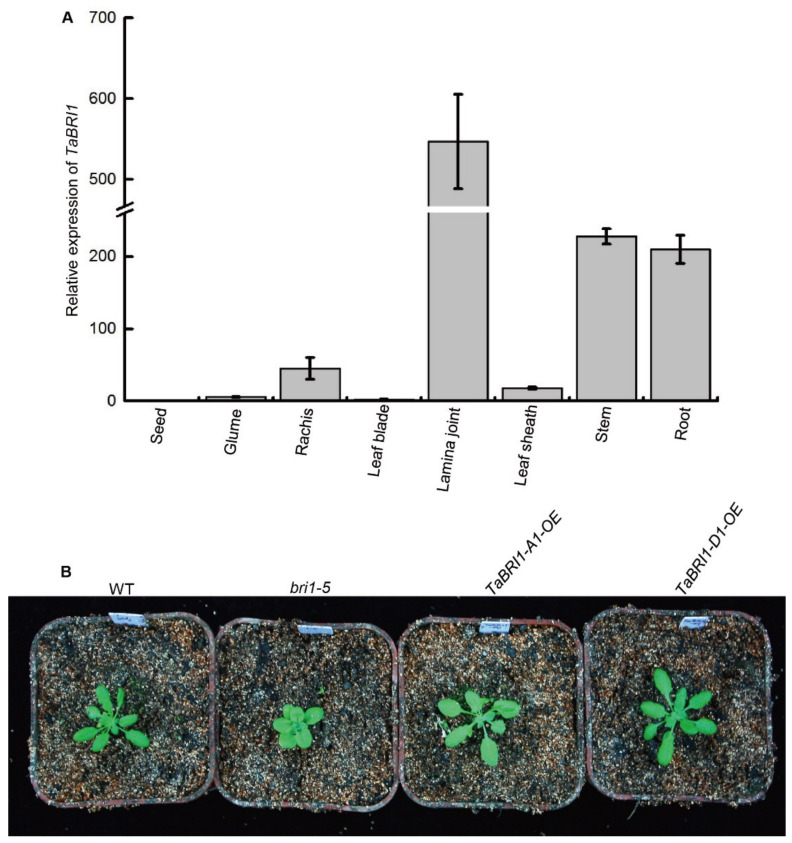
Expression pattern of *TaBRI1* gene and complementation analysis in the *Arabidopsis bri1-5* mutant by expressing *TaBRI1*. (**A**) Expression of *TaBRI1* in various tissues analyzed by real-time PCR. Various tissues were collected from plants at the reproductive stage. Each tissue had three biologic replicates and each biologic replicate comprised a pool of five plants; (**B**) complementation analysis in the *Arabidopsis bri1-5* mutant by expressing *TaBRI1-A1* and *-D1*, respectively.

Real-time RT-PCR analysis showed that *TaBRI1* was universally expressed in all tissues, with relatively high expression in lamina joint, stem, root and rachis (Figure 2A). To investigate the biology function of *TaBRI1*, we overexpressed *TaBRI1* in the *bri1-5* mutant, an ideal material for *BRI1* complementation studies, due to it is a weak mutant allele of *Arabidopsis BRI1*, semi-dwarf and setting nearly normal amount of seeds, compared to null alleles of *bri1* mutants. As shown in the Figure 2B, the transgenic plants exhibited nearly complete complementation of the *bri1-5* mutant phenotypes, resembling the phenotype of the WT. This indicated that there was functional conservation of *BRI1* between the monocot plant, wheat and the dicot plant, *Arabidopsis*.

### 3.2. TaBRI1-A1 or TaBRI1-D1 Deletion Mutants with a Decreased TaBRI1 Expression Exhibited Relatively Erect Leaves

**Figure 3:**
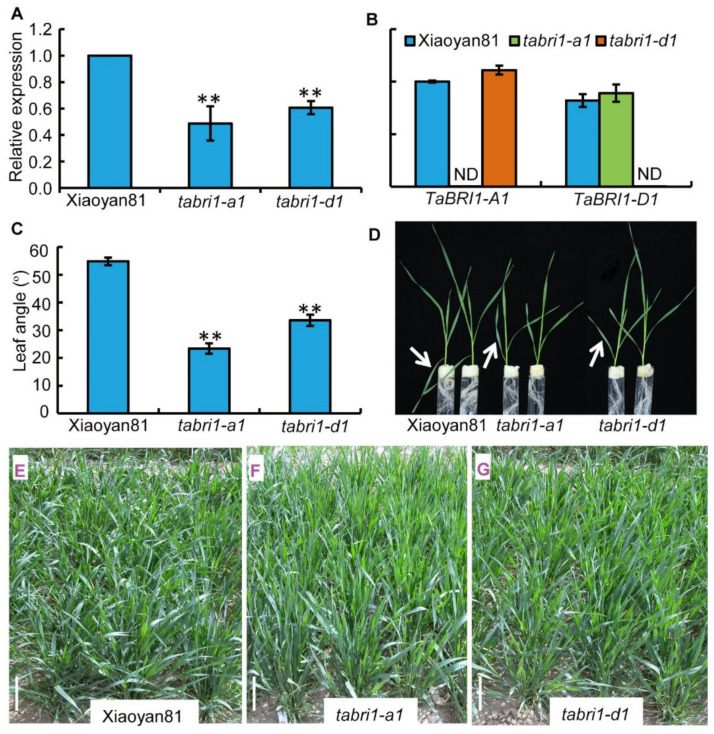
Angle between leaf blade and leaf sheath of wild-type (WT) Xiaoyan81 and *TaBRI1* mutants at seedling stage. (**A**) Relative expression levels of total *TaBRI1* genes in WT and *TaBRI1* mutants at seedling stage. Each genotype had three biologic replicates, and each biologic replicate comprised a pool of five plants. ** indicates significant differences between WT and *TaBRI1* mutants at *p* < 0.01; (**B**) relative expression levels of *TaBRI1-A1* and *-D1*. Each genotype had three biologic replicates and each biologic replicate comprised a pool of five plants; (**C**) leaf angle in WT and *TaBRI1* mutants. Each genotype had three biologic replicates, and each biologic replicate comprised fifteen plants; (**D**) Leaf morphology. The leaf of WT (left) is bent at the lamina joint indicated by the white arrow, whereas the leaves of *TaBRI1-A1* (center) and *TaBRI1-D1* (right) mutants are more erect; (**E**–**G**) morphology of WT, *TaBRI1-A1* and *TaBRI1-D1* mutants in the field at the seedling stage. Bars = 10 cm.

We isolated the *TaBRI1* deletion mutants from the ion beam-induced mutants of variety Xiaoyan81. By fragment analysis of PCR products, putative homozygous mutants lacking *TaBRI1-A1* and *-D1* were detected in two (K51 and L75) and one (B33) independent M_2_ families, respectively. Before further analysis, homozygous *TaBRI1* mutants (*TaBRI1-A1*, K51; *TaBRI1-D1*, B33) were backcrossed with their WT progenitor Xiaoyan81. The homozygous deletion lines BC_1_ F_2_ were used to analyze the expression level of *TaBRI1*. The qRT-PCR results showed that the *TaBRI1* expression was significantly reduced in the *TaBRI1-A1* or *TaBRI1-D1* deletion mutants using primers designed from conserved regions of *TaBRI1* homologous genes, compared with WT, Xiaoyan81 (Figure 3A). *TaBRI1-A1* and *-D1* transcripts were not detected in their corresponding mutants (Figure 3B), suggesting that they were deleted in their corresponding mutants.

**Figure 4:**
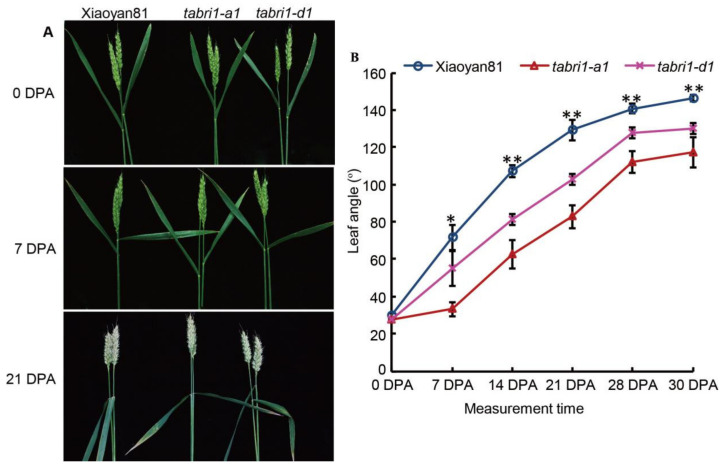
Kinetic comparison of leaf angle between the flag leaf blade and its sheath of WT and *TaBRI1* mutants at the post-anthesis stage. (**A**) Flag leaf morphology; (**B**) leaf angle in WT and *TaBRI1* mutants during the whole post-anthesis period. Each genotype had three biologic replicates, and each biologic replicate comprised fifteen plants. * and ** indicate significant differences between WT and *TaBRI1* mutants at *p* < 0.05 and *p* < 0.01, respectively.

In *TaBRI1* knock-down mutants, the second leaf angle was significantly decreased compared with that of WT at the seedling stage. The angles of the second leaf of WT, *TaBRI1-A1* and *TaBRI1-D1* were averaged 55 ± 1°, 23 ± 2° and 34 ± 2°, respectively (Figure 3C,D). Previous studies have shown that the rice *bri1* mutant of brassinosteroid receptor exhibited an erect leaf phenotype [6]. To further clarify the effect of *TaBRI1-A1* or *TaBRI1-D1* deletion on plant architecture in wheat, we tracked the plant type of the mutant from wheat seedling to the maturity in the field. The results showed that at the early jointing stage, the plant architecture of *TaBRI1-A1* mutant was compact, compared that of WT Xiaoyan81 which was loosed (Figure 3E,F). The plant architecture of *TaBRI1-D1* mutants showed weak morphologic phenotypes with less erect leaves (Figure 3G). The flag leaf of WT Xiaoyan81 possesses the characteristic of changing from erect to droopy posture. Consistent with this, in our study the flag leaf angle of Xiaoyan81 was 30° at flowering stage (0 DPA) and increased to 72° at 7 DPA (Figure 4A,B). At 14 DPA, the flag leaf of Xiaoyan81 exhibited droopy posture with 146° of the leaf angle at maturity (30 DPA) (Figure 4A,B). The increasing leaf angle with plant growth was also observed on the flag leaves of *TaBRI1-A1* and *TaBRI1-D1* mutants, however, the mutants exhibited slower increasing rates than Xiaoyan81 did during the whole post-anthesis period (Figure 4A,B). Consequently, until the late post-anthesis stage (21 DPA–28 DPA), the flag leaves of *TaBRI1* mutants change into flat posture and at maturity (30 DPA) the leaf angle was 110° to 129°, exhibiting significant reduction compared to that in WT (Figure 4A,B).

### 3.3. TaBRI1 Knock-Down Mutants Showed a Reduction in Grain Yield

**Figure 5:**
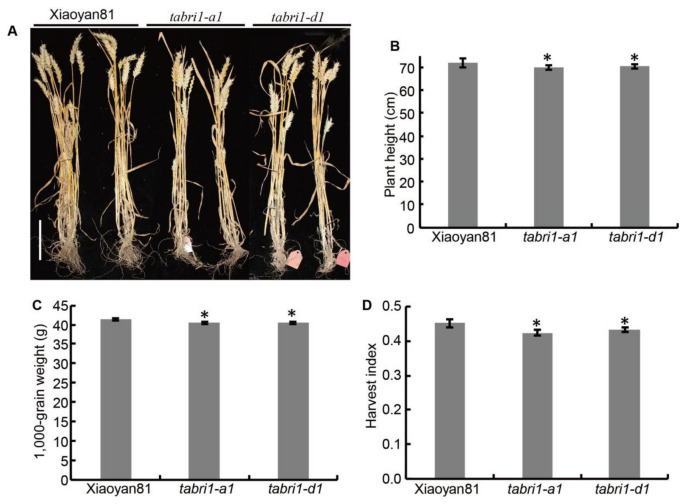
The yield traits in WT, *TaBRI1-A1* and *TaBRI1-D1* mutants. (**A**) Phenotypes of *TaBRI1-A1* and *TaBRI1-D1* mutants at maturity. Bar = 15 cm; (**B**) plant height; (**C**) 1000-grain weight; (**D**) Harvest index. All phenotypic data were measured from field-grown plants under normal cultivation conditions. Each genotype had three biologic replicates, and each biologic replicate comprised fifteen plants. * indicates significant differences between WT and *TaBRI1* mutants at *p* < 0.05.

Yield performance of *TaBRI1* knock-down mutants, *TaBRI1-A1* and *TaBRI1-D1* in the field was further analyzed. The *TaBRI1* knock-down mutants showed a significant reduction in plant height (Figure 5A,B). However, no significant differences were observed in the spike number per plant and number of grains per spike between WT and *TaBRI1* knock-down mutants, there is a significant reduction in the 1000-grain weight (TGW), leading to a decrease in grain yield per plant (Figure 5C, Supplementary Materials Figure S4). In addition, *TaBRI1* knock-down mutants had a significant reduction in the harvest index compared with WT (Figure 5D). Overall, these observations indicated that *TaBRI1* modifies plant height and reduces TGW.

### 3.4. TaBRI1 Knock-Down Mutants Showed a Reduction in Photosynthetic Rate

**Figure 6:**
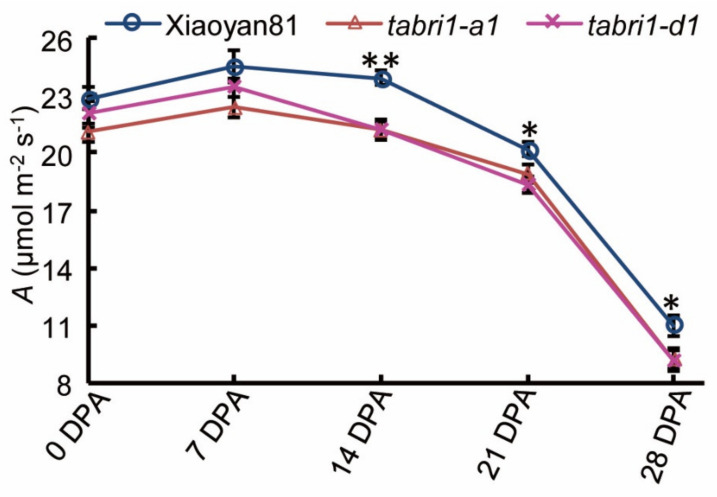
Photosynthesis parameters in WT, *TaBRI1-A1* and *TaBRI1-D1* mutants during the whole post-anthesis period. CO_2_ assimilation rate, A. Each genotype had three biologic replicates at each measurement time, and each biologic replicate comprised eight plants. * and ** indicate significant differences between WT and *TaBRI1* mutants at *p* < 0.05 and *p* < 0.01, respectively.

The flag leaf is generally considered as the major source of photosynthetic products in panicles as flag leaves after flowering produced over 50% carbohydrates that accumulate in grains of cereals such as rice [42,43,44,45]. To explore the reason for the reduction of TGW, net photosynthesis in the flag leaves of WT, *TaBRI1-A1* and *TaBRI1-D1* were measured during the grain filling stage. The results showed that *TaBRI1* knock-down mutants, *TaBRI1-A1*, *TaBRI1-D1* generally had lower CO_2_ assimilation rate than the WT (Figure 6). At the flowering stage (0 DPA) and 7 DPA, the CO_2_ assimilation rate in the *TaBRI1-A1* mutants was significantly decreased, compared with that in WT. However, there was no significant difference between the *TaBRI1-D1* and WT at the above stages. At 14 DPA, the *TaBRI1-A1* and *TaBRI1-D1* mutants both exhibited significantly lower CO_2_ assimilation rate than WT. After 14 DPA, the CO_2_ assimilation rate of WT and *TaBRI1* knock-down mutants started to decrease, but the *TaBRI1* knock-down mutants exhibited faster decreasing rates than the WT did, consequently the *TaBRI1* knock-down mutants had significantly lower CO_2_ assimilation rate than WT (Figure 6).

### 3.5. TaBRI1 Knock-Down Plants Generated by TaBRI1-A1, TaBRI1-D1 Deletion or Using Virus-Induced Gene Silencing Exhibited the Reduced Tolerance to High Light and High Temperature Stresses

**Figure 7:**
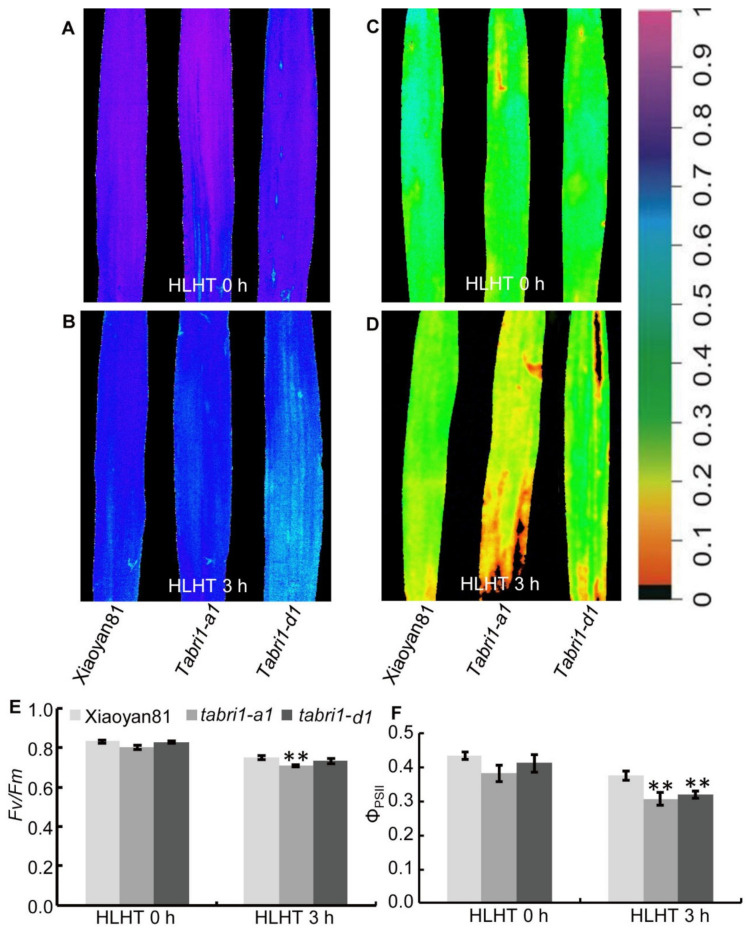
Chlorophyll fluorescence parameters of detached flag leaves in WT and *TaBRI1* mutants at 14 DPA under HLHT 3 h. (**A**–**D**) Images of the maximum photochemical efficiency (*Fv*/*Fm*) (**A**,**B**), the actual PSII efficiency (Φ_PSII_) (**C**,**D**) of WT and *TaBRI1* mutants under high light and high temperature (HLHT) 3 h. The color in (**A**–**D**) represents the value of each parameter in the color scale. (**E**,**F**) Statistic analysis of *Fv*/*Fm*, Φ_PSII_ in (**A**–**D**), respectively. Data from three biologic replicates, and each biologic replicate comprised eight plants. ** indicates significant differences between WT and *TaBRI1* mutants at *p* < 0.01.

**Figure 8:**
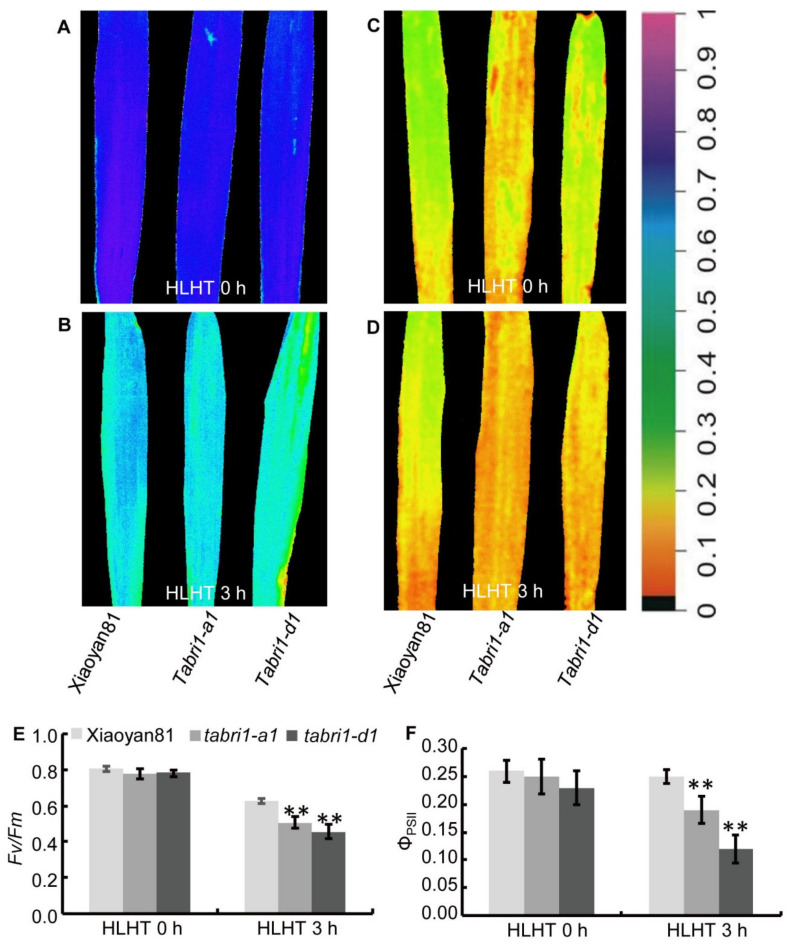
Chlorophyll fluorescence parameters of detached flag leaves in WT and *TaBRI1* mutants at 21 DPA under HLHT 3 h. (**A**–**D**) Images of the maximum photochemical efficiency (*Fv*/*Fm*) (**A**,**B**), actual PSII efficiency (Φ_PSII_) (**C**,**D**) of WT and *TaBRI1* mutants under HLHT 3 h. The color in (**A**–**D**) represents the value of each parameter in the color scale; (**E**,**F**) statistical analysis of *Fv*/*Fm*, Φ_PSII_ in (**A**–**D**), respectively. Data are from three biologic replicates, and each biologic replicate comprised eight plants. ** indicates significant differences between WT and *TaBRI1* mutants at *p* < 0.01.

Wheat plants often suffer high light and high temperature (HLHT) stresses at the late grain filling stage in Northern China Plain. High light accompanied with other adverse environments such as high temperature and/or drought will eventually lead to irreversible photooxidation of photosynthetic apparatus which will decrease photosynthetic function [46]. We further explored the role of *TaBRI1* in wheat response to HLHT stresses. The chlorophyll fluorescence parameters in the detached flag leaves were compared between WT and *TaBRI1* knock-down mutants, *TaBRI1-A1* and *TaBRI1-D1*, under HLHT stresses at 14 and 21 DPA. At 14 and 21 DPA, WT and *TaBRI1* knock-down mutants showed similar values of maximum photochemical efficiency (*Fv*/*Fm*) and actual photochemical efficiency of PSII (Φ_PSII_) before high light and high temperature (HLHT 0 h); after the detached flag leaves were treated with HLHT for 3 h (HLHT 3 h), the values of *Fv*/*Fm* and Φ_PSII_ were reduced and exhibited lower levels in the *TaBRI1* knock-down mutants than in the WT (Figure 7 and Figure 8). At 21 DPA, more obvious reduction in the *Fv*/*Fm* and Φ_PSII_ values were observed in *TaBRI1* knock-down mutants, compared with that in WT (Figure 8). The above results indicated that *TaBRI1* is required for resistance to HLHT stresses in wheat.

**Figure 9:**
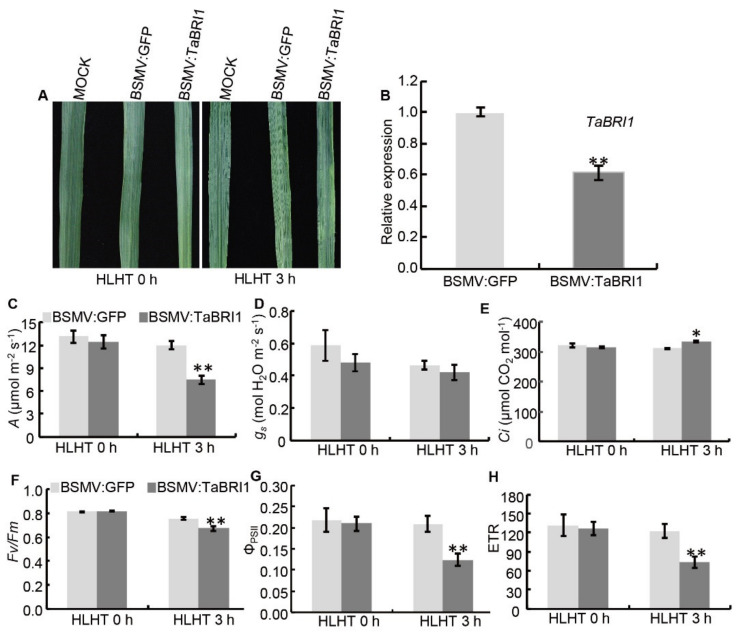
Responses to high light and high temperature stresses for 3 h (HLHT 3 h) of *TaBRI1*-silenced plants generated by barley stripe mosaic virus (BSMV)-induced gene silencing in common wheat cv. Xiaoyan 54. (**A**) Leaves exhibiting mild chlorotic mosaic symptoms were treated with HLHT 3 h at 28 days post- inoculation (dpi) with BSMV:GFP or BSMV:*TaBRI1*; (**B**) expression levels of *TaBRI1* in the BSMV:GFP and BSMV:*TaBRI1* plants. Collection of the fifth leaf from eight plants of 28 dpi was used for real-time PCR analysis. ** indicates significant differences between BSMV:*TaBRI1* and BSMV:GFP at *p* < 0.01; (**C**–**H**) *TaBRI1* silencing in common wheat cv. Xiaoyan 54 reduces the tolerance to HLHT stresses. CO_2_ assimilation rate, A (**C**), stomatal conductance, gs (**D**), intercellular CO2 partial pressure, Ci (**E**), maximal efficiency of PSII photochemistry (*Fv*/*Fm*) (**F**), the actual PSII efficiency (Φ_PSII_) (**G**), electron transport rates (ETRs) (**H**) of BSMV:GFP and BSMV:*TaBRI1* plants treated with HLHT 3 h. Data from three biologic replicates, and each biologic replicate comprised eight plants. * and ** indicate significant differences between BSMV:*TaBRI1* and BSMV:GFP plants at *p* < 0.05 and *p* < 0.01, respectively.

To further confirm the role of *TaBRI1* in wheat responses to HLHT stresses, the barley stripe mosaic virus (BSMV)-based virus-induced *TaBRI1* silencing (VIGS) technique was used. The plants exhibiting significant BSMV infection symptoms were further cultured for three weeks and treated under HLHT 3 h (Figure 9A). The leaves with non-obvious BSMV symptoms were chosen to be measured the photosynthetic parameters (A, Gs and Ci) and the efficiency of PSII (*Fv*/*Fm*, Φ_PSII_ and ETRs) to eliminate the interference of the visible bleached leaves due to the plant immunity to virus (Figure 9A). The expression level of *TaBRI1* in the BSMV:*TaBRI1* plants was significantly decreased compared that in the BSMV:GFP plants, indicating that *TaBRI1* was effectively silenced (Figure 9B). Under HLHT stresses for 3 h, CO_2_ assimilation rate, stomatal conductance (*gs*), intercellular CO_2_ partial pressure in the BSMV:*TaBRI1* plants were significantly lower than those in the BSMV:GFP plants (Figure 9C–E). Meanwhile, the *Fv*/*Fm*, Φ_PSII_ and ETRs of PSII efficiency parameters in the BSMV:*TaBRI1*-infected plants decreased by 11.1%, 40.4% and 40.4%, respectively compared with those in BSMV:GFP plants under HLHT 3 h (Figure 9F–H). In contrast, no significant differences were observed in the photosynthetic parameters (A, Gs and Ci) and the efficiency of PSII (*Fv*/*Fm*, Φ_PSII_ and ETRs) between the BSMV:GFP and BSMV:*TaBRI1* plants under normal conditions (Figure 9C–H).

### 3.6. TaBRI1 Knock-Down Mutants Is Less Sensitive to Exogenous BR Treatment than Wild Type Plants

**Figure 10:**
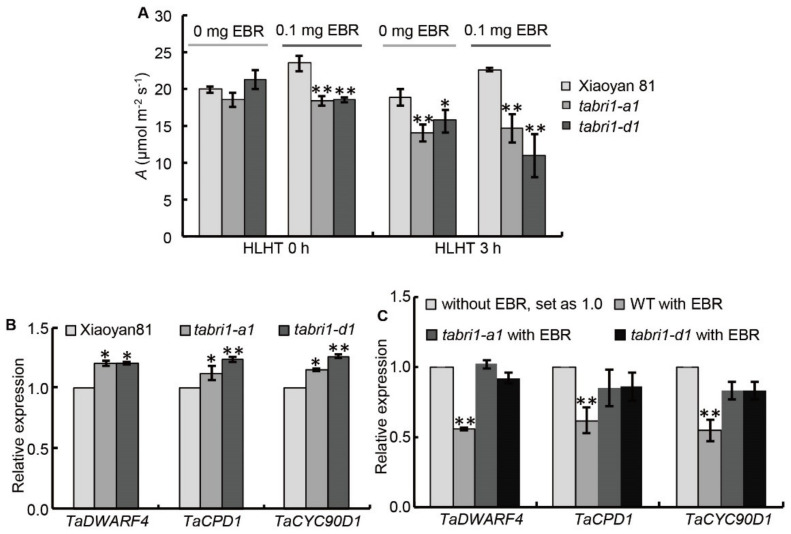
Effects of EBR on CO_2_ assimilation rate and BR-related gene expression of *TaBRI1* mutants under both HLHT 0 h and 3 h. (**A**) Effects of EBR on CO_2_ assimilation rate, A, of WT and *TaBRI1* mutants under HLHT 3 h. Data from three biologic replicates, and each biologic replicate comprised eight plants. * and ** indicate significant differences between WT and *TaBRI1* mutants at *p* < 0.05 and *p* < 0.01, respectively; (**B**) expression of BR biosynthetic genes in WT and *TaBRI1* mutants. Gene expression levels in WT are set as 1.0. Data from three biologic replicates, and each biologic replicate comprised a pool of five plants. * and ** indicate significant differences between WT and *TaBRI1* plants at *p* < 0.05 and *p* < 0.01, respectively; (**C**) expression of BR biosynthetic genes in WT and *TaBRI1* mutants with or without EBR treatment. Gene expression levels in WT without EBR treatment are set as 1.0. Data from three biologic replicates, and each biologic replicate comprised a pool of five plants. ** indicate significant differences between the control and EBR treated plants in WT at *p* < 0.01.

To test whether *TaBRI1* knock-down mutants, *TaBRI1-A1* and *TaBRI1-D1* were less sensitive to BRs than WT plants, wheat seedlings were treated with 0.1-mg/L EBR and then were subjected to HLHT stresses. As shown in Figure 10A, the CO_2_ assimilation rate of the WT seedlings was significantly increased by exogenously applied EBR under both normal conditions and HLHT stresses for 3 h. However, such increase effect was not observed in the CO_2_ assimilation rate of the *TaBRI1* knock-down mutant seedlings (Figure 10A). Considering BR signaling mutants usually have defects in feedback regulation on BR biosynthesis genes, we treated *TaBRI1* knock-down mutants with or without EBR and isolated the RNA for quantitative RT-PCR analysis of BR biosynthesis gene expression, including *TaDWARF4*, *TaCPD1* and *TaCPD90D1*. The results showed that the BR biosynthetic genes were significantly increased in the *TaBRI1* mutants (Figure 10B). Moreover, while EBR treatment reduced the expression of these genes in WT, their expression is not decreased or decreased to a lesser extent in *TaBRI1* knock-down mutants (Figure 10C). The above results suggest that there are defects in feedback regulation on BR biosynthetic genes in the *TaBRI1* knock-down mutants.

## 4. Discussion

Although the BR signal transduction pathway in the dicot *Arabidopsis* and the monocot rice have been extensively studied, the understanding of BR signal transduction in wheat is poor. In this study, we identified *TaBRI1-A1*, -*B1* and *-D1* genes and functionally characterized *TaBRI1* knock-down plants, *TaBRI1-A1* and *TaBRI1-D1*, isolated from the ion beam-induced mutants of variety Xiaoyan81 which are deletion of *TaBRI1-A1* and *-D1*, respectively. The *TaBRI1* knock-down plants exhibited relatively erect leaves and a significant reduction in the 1000-grain weight. We further dissected the underlying physiological mechanisms by measuring the photosynthetic capacity and assessing the tolerance ability to highlight and high temperature (HLHT) stresses often suffered by wheat in Northern China Plain at the late grain filling stage.

### 4.1. Knock-Down the Expression of TaBRI1 Reduced Leaf Angles and the 1000-Grain Weight

Leaf angle, the degree of bending between the leaf blade and leaf sheath is an important factor which directly affects crop architecture and grain yields [47]. Erect leaves are desired to avoid shade and capture more light for photosynthesis when plants are grown at high planting density, all of which increase crop yields [31,32,48]. The erect leaf phenotype of *TaBRI1-A1* and *TaBRI1-D1* mutants can be observed as early as the seedling stage both of which showed a significant reduction in the *TaBRI1* expression (Figure 3). The *TaBRI1* knock-down mutants, *TaBRI1-A1* and *TaBRI1-D1*, displayed a significant decrease in the leaf angle during the whole grain filling stage (Figure 4). *d61-1* and *d61-2*, loss-of-function mutants of *OsBRI1*, showed insensitivity to BR, erect leaves and dwarf culms [6]. Similarly, barley mutant of *HvBRI1*, *uzu*, exhibited a semi-dwarf and upright plant architecture [33]. The plant height of *TaBRI1-A1* and *TaBRI1-D1* mutants decreased by 2.7% and 1.9%, respectively compared with that of WT (Figure 5B). This reduction was far less than those in monocot plants, rice and barley. The main reason may be that BR receptor is encoded by multiple genes and has functional redundancy due to wheat as a polyploid plant. In addition, the *TaBRI1-A1* and *TaBRI1-D1* mutants did not show serious growth and development deficiencies which was either supposedly resulted from functional redundancy (Figure 4). The leaf angle and plant height of *TaBRI1-A1* mutant was slightly lower than that of *TaBRI1-D1* mutant (Figure 3C–G and Figure 4). This may be due to the functional differences in *TaBRI1-A1* and *-D1* proteins or differences in gene expression. In consistent, we found the expression level of total *TaBRI1* was lower in *TaBRI1-A1* mutant than that in the *TaBRI1-D1* mutant (Figure 3A).

The grain yield of loss-of-function and brassinosteroid-deficient mutants such as *d61* (mutant of OsBRI1), *brd1* (*brassinosteroid-deficient dwarf1*) and *d2* (defective in *OsDWARF*) decreased because of morphologic alterations in their reproductive development [6,25,49]. However, no significant differences were observed in the spike number per plant and number of grains per spike between WT and *TaBRI1-A1* and *TaBRI1-D1* mutants (Supplementary Materials Figure S4). In contrast, a significant reduction in the 1000-grain of *TaBRI1* knock-down mutants were observed (Figure 5C). Previous studies have shown that the combination of erect leaves and dense planting can improve crop yield in BR-related mutants [31,50]. The *TaBRI1* knock-down mutants, *TaBRI1-A1* and *TaBRI1-D1* showed erect leaves with a slight reduction in grain yield per plant. Thus, it was valuable to further explore whether *TaBRI1* mutants have potentiality to increase crop yield in breeding under dense planting.

### 4.2. Knock-Down the Expression of TaBRI1 Reduced Photosynthetic Rate and the Tolerance to High Light and High Temperature Stresses

During the flowering and grain-filling stages, the CO_2_ assimilation rate in the flag leaf of *TaBRI1* knock-down mutants, *TaBRI1-A1* and *TaBRI1-D1* was lower than that of WT, suggesting that BR receptor *TaBRI1* was involved in the regulation of photosynthesis in wheat (Figure 6). Photosynthetic products contributed more than 90% of crop biomass [51]. In addition, when other genetic factors are not altered, there was a close relationship between enhanced photosynthesis, biomass and yield [52]. Thus, it may be a possible reason for the significant reduction in the 1000-grain that the decreased CO_2_ assimilation rate during the whole grain filling stage in *TaBRI1* knock-down mutants (Figure 5C and Figure 6). Recent study showed that autophosphorylation inhibition of the tyrosine-831 in BRI1 receptor kinase could increase the maximum carboxylation rate of Rubisco and promote the regeneration of RuBP, leading to an enhancement of shoot growth [53]. It remains further to be studied how the photosynthesis is regulated by BR signal transduction in the *TaBRI1* knock-down mutants. Kim et al. (2010) found that some genes encoding chloroplast-localized proteins were downregulated in the cold-resistant *bri1* mutant [54]. Therefore, it was either believed that the lack of chloroplast assembly, especially the assembly of light system II, possibly led to the decrease of photosynthetic rate in *TaBRI1* knock-down mutants.

High light at noon in sunny days often inhibits CO_2_ assimilation rate in flag leaves, thus leading to approximately 10% loss of potential carbon assimilation under natural conditions [55]. Other adverse environments such as high temperature and/or drought during the grain filling period will eventually change the reversible photoinhibition into irreversible photooxidation of photosynthetic apparatus which will result in a decrease in photosynthetic function [45]. Wheat plants often suffer high light and high temperature (HLHT) stresses at the late grain filling stage in Northern China Plain. Thus, we speculated there may be some relationship between the tolerance to HLHT stresses and the decrease of photosynthetic function in the *TaBRI1* knock-down mutants. In our study, it was indeed found that the photosynthetic rate and maximum photochemical efficiency (*Fv*/*Fm*) in *TaBRI1-A1* and *TaBRI1-D1* mutants were obviously lower than those in WT Xiaoyan81 after HLHT three hours, indicating the *TaBRI1-A1* and *TaBRI1-D1* mutants are more sensitive to HLHT stresses than WT (Figure 7, Figure 8, Figure 9 and Figure 10A). In consistent, previous studies showed that overexpression of *TaBRI1* in *Arabidopsis* showed thermo tolerance phenotype at the seedling stage with increased chlorophyll content, photosystem II activity and membrane stability [56]. However, the *bri1-9* mutant, a dwarf mutant caused by defective BR signaling, exhibited an increased tolerance to cold stress due to it could always be alert to stresses by constitutive activation of subsets of defense [54]. Different from *bri1-9* mutant tolerant to cold stress, *TaBRI1-A1* and *TaBRI1-D1* mutants were not resistant to HLHT stresses possibly due to the lack of constitutive activation of defense responses. The other possible reason may be the difference in defense mechanism of cold stress and HLHT stresses. We also observed that foliar spraying EBR significantly increased the photosynthetic rate and the tolerance to HLHT stresses in WT Xiaoyan81. Compared with that, EBR spraying had no effects on the photosynthetic rate and the tolerance to HLHT stresses in *TaBRI1-A1* and *TaBRI1-D1* mutants. Moreover, the photosynthetic rate of *TaBRI1-A1* and *TaBRI1-D1* mutants was decreased by spraying EBR under HLHT stresses (Figure 10A). Yu et al. (2004) demonstrated that 10^−8^ M or 10^−7^-M EBR effectively improve photosynthetic rate and high concentration of EBR has no effect on photosynthetic efficiency [57]. Therefore, excessive concentration of EBR treatment has no favor to plant resistance to stresses. The BR biosynthetic genes were significantly increased under normal conditions and is not decreased or decreased to a lesser extent after EBR treatment in *TaBRI1* knock-down mutants due to the defects in feedback regulation on BR biosynthesis genes (Figure 10B,C). Supposedly, *TaBRI1* knock-down mutants may accumulate more BR, thus 0.1-mg/L EBR application may be too high for the mutants to confer the resistance to HLHT stresses.

## 5. Conclusions

In summary, we found exogenous BR treatment induced the tolerance to high light and high temperature stresses in wheat seedlings. To explore the detailed mechanisms of BR signaling in high light and high temperature stresses response in wheat. We cloned three *BRI1* homologous genes *TaBRI1-A1*, *-B1* and *-D1* from hexaploid wheat (*Triticum estivum* L.) and further isolated the *TaBRI1* knock-down mutants from the ion beam-induced mutants of variety Xiaoyan81, *TaBRI1-A1* and *TaBRI1-D1*. *TaBRI1* knockdown plants generated by *TaBRI1-A1*, *TaBRI1-D1* deletion or using virus-induced gene silencing exhibited the reduction in the photosynthetic parameters (A, Gs and Ci) and the efficiency of PSII (*Fv*/*Fm*, ΦII and ETR) especially under high light and high temperature stresses. The *TaBRI1* knock-down mutants exhibited relatively erect leaves and a significant reduction in the 1000-grain weight probably because of the reduced photosynthetic rate and tolerance to high light and high temperature stresses. Further efforts will be directed to understand the detailed molecular mechanisms of *TaBRI1* and the signaling pathway involved in regulating these processes.

## Supplementary Materials

The following are available online at https://www.mdpi.com/2223-7747/9/7/840/s1, Figure S1: Multiple alignment of deduced amino acid sequence of *TaBRI1* of A (*TaBRI1-A1*), B (*TaBRI1-B1*) and D (*TaBRI1-D1*) genomes; Figure S2: The leaf angle in the wild type (WT), *TaBRI1* mutants and corresponding BC_1_ F_2_ homozygous individuals for *TaBRI1* deletion identified with *TaBRI1-A1* and *TaBRI1-D1* specific primers at 14 DPA; Figure S3: The root length of WT and *TaBRI1* mutants at seedling stage; Figure S4: The yield traits in WT, *TaBRI1* mutants; Figure S5: Photosynthesis parameters in WT and *TaBRI1* mutants during the whole post-anthesis period; Table S1: Primers used in this work.
