# Supplementary material for: Knock-Down the Expression of Brassinosteroid Receptor TaBRI1 Reduces Photosynthesis, Tolerance to High Light and High Temperature Stresses and Grain Yield in Wheat
Source: Plants (Basel). 2020 Jul 3;9(7):840. doi: 10.3390/plants9070840 (PMC7411796; doi:10.3390/plants9070840)
Supplement: Supplementary file 1 [file plants-09-00840-s001.pdf]

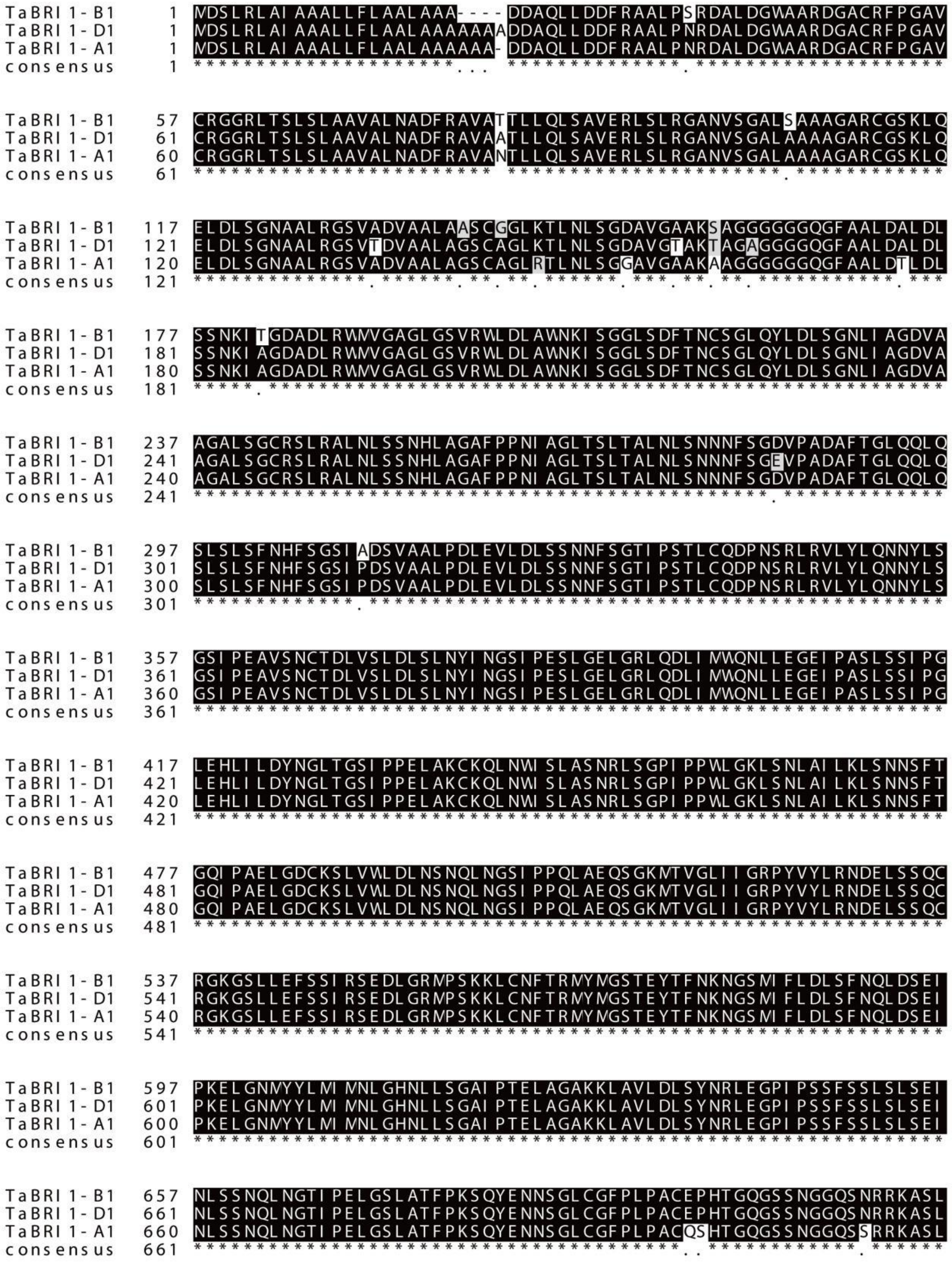

Figure S1. Multiple alignment of deduced amino acid sequence of TaBRI1 of A (TaBRI1-A1), B (TaBRI1-B1) and D (TaBRI1-D1) genomes. Black regions show identical amino acid residues among the three proteins, and the gray regions show identical amino acid residues among two of the three proteins. The points show the absent amino acid residues. The positions of amino acids are given on the right.

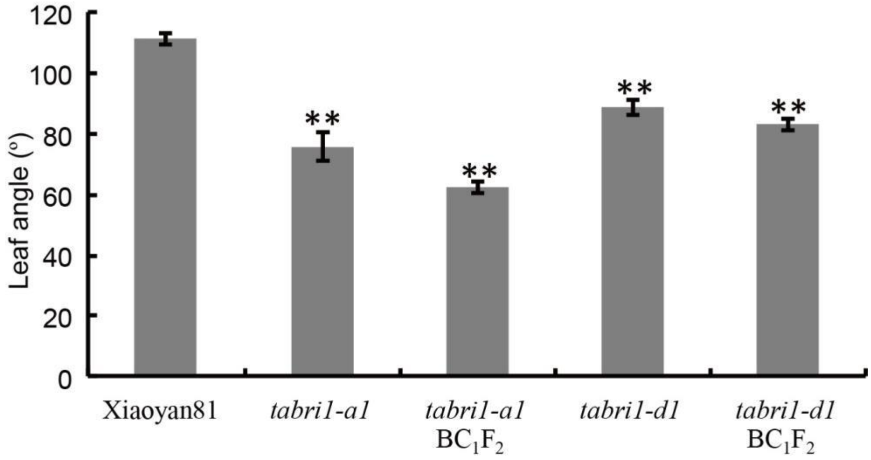

Figure S2. The leaf angle in the wild type (WT), *tabri1* mutants, and corresponding. BC_1F_2 homozygous individuals for *tabri1* deletion identified with *TaBRI1-A1* and *TaBRI1-D1* specific primers at 14 DPA. DPA represents days post-anthesis. Data are means ± SE of fifteen plants.

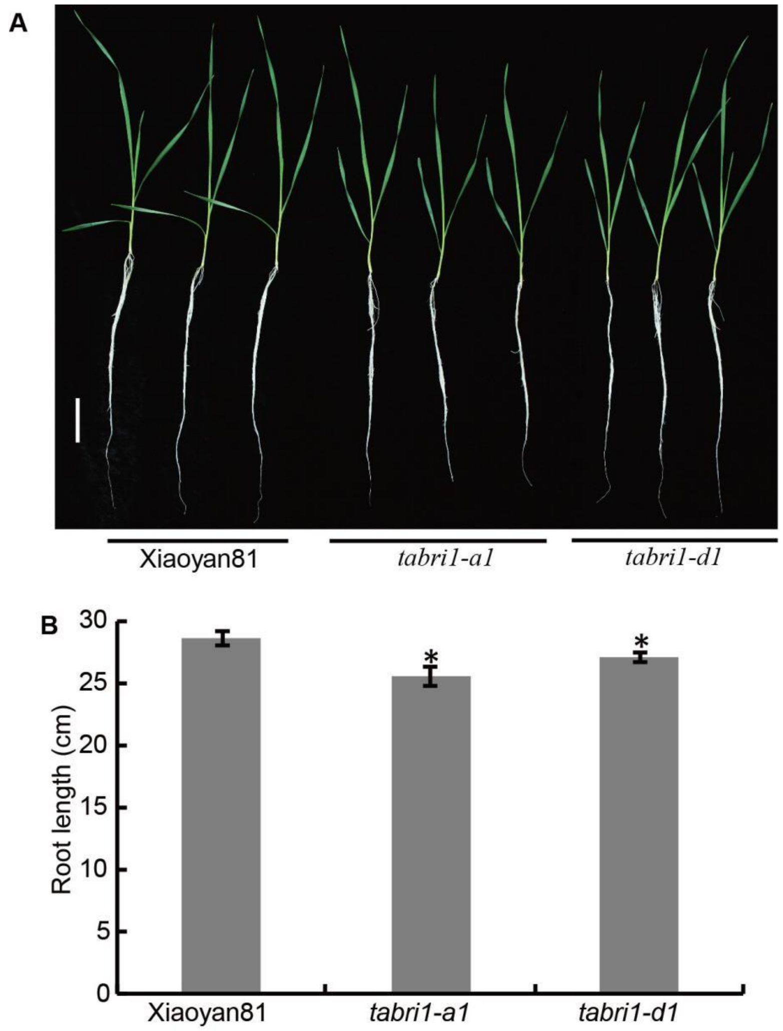

Figure S3. The root length of WT and *tabri1* mutants at seedling stage. (**A**) Root morphology in WT and *tabri1* mutants. Bar = 5 cm; (**B**) Statistic analysis of root length in WT and *tabri1* mutants. All values are means ± SE (n = 15). * indicates significant differences between WT and *tabri1* mutants at $P < 0.05$.

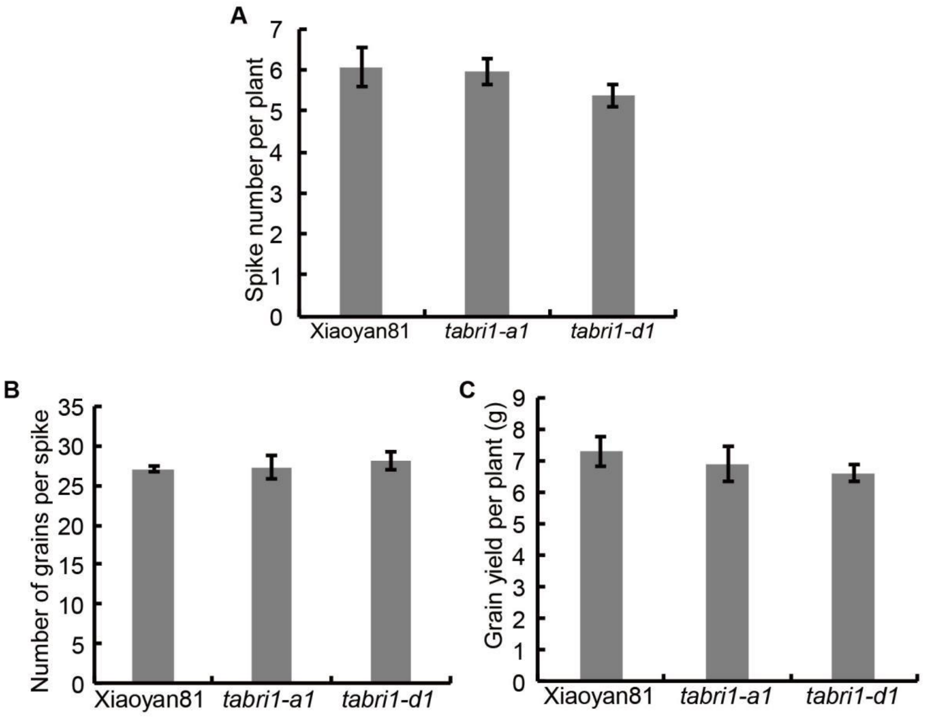

Figure S4. The yield traits in WT and *tabri1* mutants. (**A**) Spike number per plant; (**B**) Number of grains per spike; (**C**) Grain yield per plant. All phenotypic data were measured from field-grown plants under normal cultivation conditions. All values are means ± SE (n = 15).

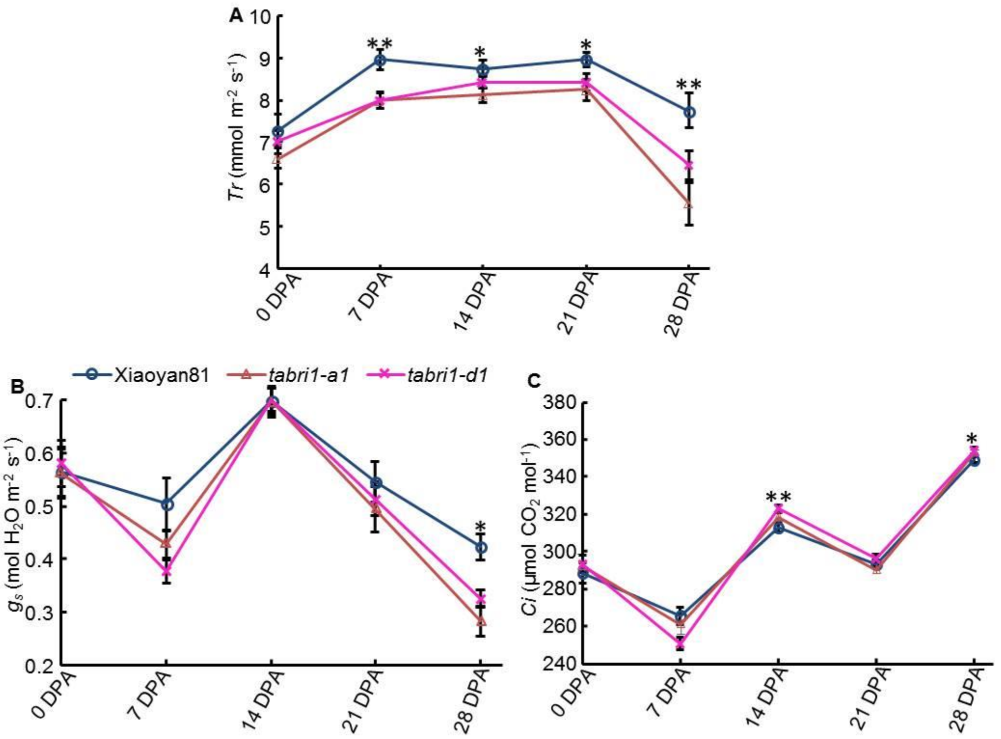

Figure S5. Photosynthesis parameters in WT and *tabri1* mutants during the whole post-anthesis period. (**A**) Transpiration rate, Tr; (**B**) Stomatal conductance, gs; (**C**) Intercellular CO_2 partial pressure, Ci. All values are means ± SE (n = 8). * and ** indicate significant differences between WT and *tabri1* deletion plants at $P < 0.05$ and $P < 0.01$, respectively.

Table S1. Primers used in this work.

Experiment	Name	Sequence
realtime PCR	TaBRI1(QP)F	GCCACCAGAGTACTACCAGAGCTTC
realtime PCR	TaBRI1(QP)R	GCGATTTTCAAATGCTCCAGC
realtime PCR	TaBRI1-A1(QP)F	CTGGAGCTGGAGCTGCTGGAG
realtime PCR	TaBRI1-A1(QP)R	TaBRI1.1specifeic(R)
realtime PCR	TaBRI1-D1(QP)F	GTGATGACGATGTTCAAGGAGATCC
realtime PCR	TaBRI1-D1(QP)R	TaBRI1.2specifeic(R)
realtime PCR	ACTINF	ACCTTCAGTTGCCCAGCAAT
realtime PCR	ACTINR	CAGAGTCGAGCACAATACCAGTTG
realtime PCR	TaDWARF4(QP)F	GGTTGCCCTAAAGCCGTTGAA
realtime PCR	TaDWARF4(QP)R	CGTTGCCCTTCCATCTCCAAG
realtime PCR	TaCPD1(QP)F	GAGATGGCAGAGCAACAACAAAC
realtime PCR	TaCPD1(QP)R	CGAGTGGTGGGAAAGAAGACGAG
realtime PCR	TaCYC90D1(QP)F	CTCGCCGTCAAGTTCCTCAG
realtime PCR	TaCYC90D1(QP)R	CATGCTGCGTGAATGACAAGGAC
VIGS	TaBRI1(VIGS)F	AGCTAGCCAGAGCTTGGTTCTC
VIGS	TaBRI1(VIGS)R	TGCTAGCGGTACTTGCCTCATC
Chromosome location	TaBRI1(RACE)F1	GCCACCAGAGTACTACCAGAGCTTC
Chromosome location	TaBRI1.1specifeic(R)	CCCGGCCCCAAACTGGATGG
Chromosome location	TaBRI1.2specifeic(R)	GGTATCAGCATCTGAGAGACTGTG
Chromosome location	TaBRI1.3specifeic(R)	CAGCTCCGGGTCAAACACATCC
TaBRI1 amplification	TaBRI1(F)	CTTCTCGCATGGTCTCAAGGTAG
TaBRI1 amplification	TaBRI1(R3)	GCGATTTTCAAATGCTCCAGC
Vector construction	TaBRI1(pri)F	GGAATTCCATATGGATTCCCTGCGGCTG
Vector construction	TaBRI1(pri)R	CGCGGATCCTAATCTTTCTCCTCCTTG
3′-RACE	TaBRI1(RACE)F1	GCCACCAGAGTACTACCAGAGCTTC
3′-RACE	TaBRI1(RACE)F2	CCGGAGCTGCTGAAGGACGATC
